# Psychometric evaluation of the German version of the Opening Minds Stigma Scale for Health Care Providers (OMS-HC)

**DOI:** 10.1186/s40359-021-00592-9

**Published:** 2021-05-21

**Authors:** Gianfranco Zuaboni, Timon Elmer, Franziska Rabenschlag, Kolja Heumann, Susanne Jaeger, Bernd Kozel, Candelaria I. Mahlke, Anastasia Theodoridou, Matthias Jaeger, Nicolas Rsch

**Affiliations:** 1 Sanatorium Kilchberg AG, Psychiatric and Psychotherapy Hospital, Alte Landstrasse 70, 8802 Kilchberg, Switzerland; 2 University of Groningen, Groningen, The Netherlands; 3 Psychiatric University Clinics Basel, Basel, Switzerland; 4 Department of Psychiatry and Psychotherapy, Medical University Brandenburg, Neuruppin, Germany; 5 Department of Psychiatry and Psychotherapy I, Ulm University, ZfP Sdwrttemberg, Ravensburg, Germany; 6 University Psychiatric Services Bern, Bern, Switzerland; 7 Department of Psychiatry and Psychotherapy, University Medical Centre Hamburg-Eppendorf (UKE), Hamburg, Germany; 8 Department of Psychiatry, Psychotherapy and Psychosomatics, University Hospital of Psychiatry Zurich, Zurich, Switzerland; 9 Psychiatrie Baselland, Liestal, Switzerland; 10 Department of Psychiatry and Psychotherapy II, Ulm University and BKH Gnzburg, Ulm/Gnzburg, Germany

**Keywords:** Attitudes, Stigma, Social distance, Therapeutic relationship, Psychometrics, Recovery, Mental illness

## Abstract

**Background:**

Healthcare professionals can be a source of stigma and discrimination for people with mental illness, and anti-stigma programs are needed for this target group. However, there is no validated German language scale to assess attitudes of healthcare professionals towards people with mental illness. This study had the aim to validate the German language version of the Opening Minds Stigma Scale for Health Care Providers (OMS-HC), a self-report measure of stigmatizing attitudes.

**Methods:**

Staff (n=392) on general psychiatric inpatient wards (excluding child, forensic and geriatric psychiatry) at five psychiatric hospitals in Switzerland (n=3) and Germany (n=2) participated in the study. The internal consistency of the OMS-HC was examined as well as its factor structure using exploratory and confirmatory factor analyses. To assess the scales concurrent validity, we used the Social Distance Scale.

**Results:**

Internal consistency for the OMS-HC total score was good (=0.74), acceptable for the subscales Attitudes (=0.62) and Social Distance (=0.69), and poor for the Disclosure subscale (=0.55). The original three-factor structure fit our data well. The OMS-HC total score and the Social Distance subscale score were significantly correlated with the Social Distance Scale, supporting concurrent validity.

**Conclusion:**

The German version of the OMS-HC demonstrated satisfactory psychometric properties and can be recommended for future research and intervention evaluation.

**Supplementary Information:**

The online version contains supplementary material available at 10.1186/s40359-021-00592-9.

## Background

Stigmatization by healthcare professionals (HCP) is widespread and has serious consequences, including poorer physical and mental healthcare for people with mental health problems (PWM) [1,3]. An explanation may be the perceived difference between HCP and PWM, i.e. that they (PWM) are completely different from us (HCP) [4, 5]. Stigma experienced or anticipated by PWM can impair the therapeutic relationship with HCPs, which in turn has a negative impact on the recovery process [6,8]. Many barriers to use healthcare services are related to stigma [9]. As a result, PWM are not taken seriously and do not receive the treatment they need, with negative health consequences and lower life expectancy than the general population [10, 11].

From a stigma perspective, mental healthcare professionals can be sources of stigma if they endorse stigmatizing attitudes. However, they can also be agents for change and allies of people with mental illness. That may especially apply to situations in which they disclose their experience of own mental health problems to colleagues, patients or others [12]. There is no evidence that attitudes towards people with mental illness are less negative among German-speaking healthcare professionals compared to other countries [13]. This highlights the need for measures to rigorously evaluate anti-stigma interventions for this key target group. However, we are not aware of a validated German language scale in this domain.

The Opening Minds Stigma Scale for Health Care Providers (OMS-HC) was developed to gauge the attitudes of HCPs towards PWM [14]. Initially a 20-item version of the OMS-HC was administered to 787 HCPs across Canada and a factor analysis yielded inconsistent findings. Another factor analysis was conducted in a larger and more representative sample with the 20-item version [15], resulting in a 3-factor solution: (1) attitudes, (2) disclosure and help-seeking, and (3) social distance as well and resulted in a briefer 15-item version. The overall internal consistency was =0.79 for the 15-item scale and =0.67 (disclosure) or =0.68 (attitudes, social distance) for the subscales.

In this study, we present the results of the psychometric examination of a German translation of the 15-item OMS-HC in terms of its internal consistency, factor structure and concurrent validity.

## Methods

### Translation procedure

The translation procedure followed recognized guidelines [16]. The translation of the original OMS-HC (Additional file 1) into German was conducted by an experienced mental health nurse. The German version was then back-translated into English by a bilingual peer worker. Together with a bilingual psychiatrist, the two translators compared and discussed the two English versions and the German translation. The revised German version was then carefully discussed with a group of mental health nurses, psychologists and psychiatrists (n=9). Based on the group discussion, minor adjustments were made to improve the clarity of the German version, resulting in the final version evaluated in this study.

### Design and participants

This study was part of a larger cross-sectional study, which was conducted among staff on general psychiatric inpatient wards (excluding child, forensic and geriatric psychiatry; n=1629) at five psychiatric hospitals in Switzerland (n=3) and Germany (n=2) [17, 18]. All HCP staff in all five hospitals that worked directly with patients were invited to participate in the study. Among participants who volunteered for this study, an online survey was conducted in each hospital, the study was approved either by the board of directors or the respective ethics committee. Participation was voluntary.

Of the eligible HCPs (n=1629), 428 (26%) participated and 397 completed the OMS-HC questionnaire. Due to missing data or implausible response patterns, five participants were excluded from the analysis and 392 remained. About one third were male, and the most common profession was mental health nursing, followed by psychiatrists and psychologists (Table 1).

**Table 1:** Sample characteristics

Gender	n	%
Female	244	62.2
Male	148	37.8
*Age*		
1825years	35	8.9
2635years	130	33.2
3645years	95	24.2
4655years	97	24.7
>55years	35	8.9
*Profession*		
Mental health nurse	260	66.3
Physician	49	12.5
Psychologist	23	5.9
Social worker	16	4.1
Other (occupational therapist, art therapist)	44	11.2

### Measures

#### Opening Minds Stigma Scale for Health Care Providers

Participants completed online the German version of the Opening Minds Stigma Scale for Health Care Providers (OMS-HC). It is a self-report questionnaire with 15 items (e.g. There is little I can do to help people with mental illness), with a 5-point Likert scale (1/strongly disagree, 2/disagree, 3/neither agree nor disagree, 4/agree, 5/strongly agree). Items 2, 6, 7 and 8 are reverse-coded. The scale yields a mean score from 1 to 5, with higher scores indicating more stigmatizing attitudes. The English items can be found in Table 2 and in the Additional file 1, the German version is provided in Additional file 2.

**Table 2:** Factor analysis with varimax rotation (n=392); factor loadings>0.40 in bold

	Factors			Item-total correlation	Alpha if deleted
	1	2	3		
**Factor 1:** Attitudes of healthcare providers towards people with mental illness					
1. I am more comfortable helping a person who has a physical illness than I am helping a person who has a mental illness	**0.45**	0.16	0.19	0.49	0.72
9. Despite my professional beliefs, I have negative reactions towards people who have mental illness	**0.48**	0.23	0.10	0.48	0.72
10. There is little I can do to help people with mental illness	**0.44**	0.07	0.05	0.37	0.73
11. More than half of people with mental illness dont try hard enough to get better	0.33	0.19	0.22	0.48	0.72
13. Healthcare providers do not need to be advocates for people with mental illness	0.22	-0.04	0.25	0.39	0.74
15. I struggle to feel compassion for a person with a mental illness	**0.45**	0.06	0.33	0.51	0.72
**Factor 2:** Disclosure/help-seeking					
3. If I were under treatment for a mental illness, I would not disclose this to any of my colleagues	0.03	**0.40**	0.03	0.35	0.72
4. I would see myself as weak if I had a mental illness and could not fix it myself	0.21	**0.56**	0.08	0.49	0.72
5. I would be reluctant to seek help if I had a mental illness	0.18	**0.50**	0.03	0.47	0.73
8. If I had a mental illness, I would tell my friends^a^	0.00	0.28	0.35	0.44	0.73
**Factor 3:** Social distance					
2. If a colleague with whom I work told me they had a managed mental illness, I would be just as willing to work with him/her^a^	0.15	0.00	**0.52**	0.46	0.72
6. Employers should hire a person with a managed mental illness if he/she is the best person for the job^a^	0.05	0.06	**0.68**	0.51	0.72
7. I would still go to a physician if I knew that the physician had been treated for a mental illness^a^	0.25	0.01	**0.61**	0.56	0.71
12. I would not want a person with a mental illness, even if it were appropriately managed, to work with children	0.22	0.14	**0.47**	0.57	0.71
14. I would not mind if a person with a mental illness lived next door to me^a^	0.16	0.22	0.38	0.51	0.72

Bartletts test of sphericity (x^2^=857.25, *p*<2.2e-16) and the KaiserMeyerOlkin value was 0.811; alpha=0.74 (0.62 for factor 1, 0.55 for factor 2, and 0.69 for factor 3)

^a^Reverse-scored item

#### Social distance scale

The desire for social distance from people with mental illness was assessed by the Social Distance Scale (SDS), based on Bogardus work and frequently used by Bruce Link and his colleagues [19]. The scale includes seven items (e.g. How would you feel having someone with a severe mental illness as a neighbor?). Respondents rated each question from 0 (definitely willing) to 3 (definitely unwilling). The overall SDS score represents the mean of all seven items from 0 to 3, with higher scores indicating stronger social distance (Cronbachs alpha in our study was 0.75).

### Ethics

The cantonal ethics committee of Zurich confirmed to us in writing that the study does not fall within its area. The study was approved by the local ethics committee (Ethics Committee of the University of Ulm) and the internal ethics officer (University Medical Center Hamburg-Eppendorf). Furthermore, in all hospitals, the board of directors and the other relevant clinics internal departments (personal departments, departments of quality management and data protection commissioners) agreed with the study protocol. Participation in the study was voluntary and anonymous. The informed consent was obtained from all participants. All methods of the study were carried out in accordance with relevant guidelines.

### Statistical analysis

The internal consistency of the OMS-HC was assessed using Cronbachs alpha for the total score and each subscale. To determine the scales factor structure, we took a two-step approach. First, we explored the factor structure and factor loadings of each item in our data using exploratory factor analysis (EFA). Differences in factor loadings could result from cultural and contextual differences in the endorsement of stigma by mental health care workers. Therefore we also conducted a confirmatory factor analysis (CFA) to compare our data to the initial factor structure reported by Modgil et al. [15]. The fit indices of the CFA were evaluated with respect to existing fit criteria for CFAs [20], with good model fit indicated by CFI0.95, SRMR0.08, RMSEA0.06). Concurrent validity was examined by Pearson correlations between the OMS-HC and the SDS scale. The R Software Packages *psych* [21] and *lavaan* [22] with the WLSMV estimator were used to conduct the EFA and CFA, respectively. We used *Hmisc* [23] for the calculation of the correlations and *psych* [21] was used to assess reliability.

## Results

### Descriptives

On average, participants scored an OMS-HC total mean of 1.94 (SD=0.41). In the three subscales the following means were obtained: M=1.71 for attitudes (SD=0.45), M=1.91 for social distance (SD=0.58), and M=2.32 for disclosure and help seeking (SD=0.64). With regards to the Social Distance Scale (SDS), participants on average obtained a value of 2.21 (SD=0.79).

### Internal consistency and intercorrelations

The Cronbachs alpha for the German version of the OMS-HC scale indicated good internal consistency (=0.74), whereas internal consistency for the subscales Attitudes (=0.62) and Social Distance (=0.69) was acceptable and poor for the subscale Disclosure (=0.55). The total score of the OMS-HC correlated strongly with the subscales (attitudes: *r*=0.78, disclosure: *r*=0.68, social distance: *r*=0.78) and the subscales were moderately intercorrelated (see Table 3 for details).

**Table 3:** Pearson correlations of the OMS-HC subscales and the Social Distance Scale (SDS)

	OMS total score	OMS attitude	OMS disclosure	OMS distance
OMS attitude	0.78***			
OMS disclosure	0.68***	0.30***		
OMS distance	0.78***	0.45***	0.27***	
SDS	0.46***	0.25***	0.19**	0.58***

**p<0.01; ***p<0.001

### Factor analyses

An EFA was carried out to explore the factor structure and factor loadings in our data (Table 2). The EFA provided a three factors solution with eigenvalues of 3.49, 1.54, and 1.21.

Of the six items of factor 1 (attitudes), five had a factor loading of 0.330.48 and one had only 0.22. Of the four items of factor 2 (disclosure), three had a factor loading of 0.400.56 and one had 0.28. For factor 3 (social distance), the five items had values from 0.38 to 0.68. Item 11 showed cross-loadings on all three factors (Table 2), item 13 on factors 1 and 3, item 8 on factors 2 and 3, and item 14 on all three factors.

**Fig. 1:**
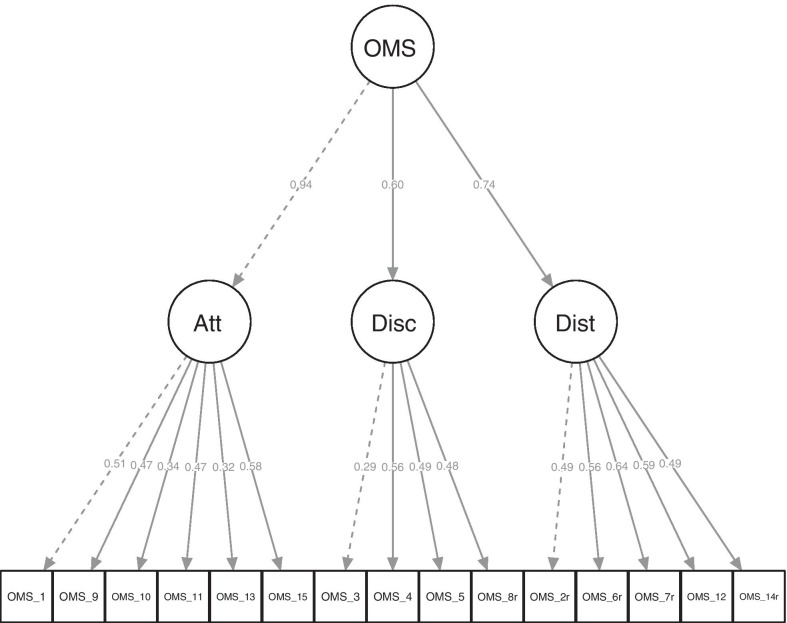
Factor loadings of the confirmatory factor analysis with three factors attitude, disclosure, and social distance. The dashed lines indicate which item was used for the scaling by fixing factor loadings. The figure was created using the sem*Plot* R-package [25]

In our CFA to examine the fit of our data to the original three-factor structure, there was a good model fit according to the criteria of Hu and Bentler [20] with ^2^(87)=149.29, *p*<0.001, comparative fit index (CFI)=0.92, standardized root mean square residual (SRMR)=0.05; root mean square error of approximation (RMSEA)=0.04; RMSEA 90% confidence interval (CI)=[0.030.05]. A significant ^2^ test does not necessarily suggest poor model fit as it is considered highly sensitive in large samples [24]. Figure1 shows factor loadings of this CFA. There are low factor loadings for some items (e.g., Item 3). These low factor loadings likely show the unique contribution of the respective item to the theoretical construct. They do not significantly worsen the model fit as they do not load better on other factors (see EFA and Table 2; e.g., Item 3 loads with 0.40 on the disclosure factor, but only with 0.03 and 0.03 on the other two).

### Concurrent validity

Pearson correlations were calculated between the OMS-HC total score, the three subscale scores and the SDS total score (Table 3). All four correlations were significantly positive, especially between SDS and the OMS-HC total and social distance subscale scores.

## Discussion

Our results provide evidence for good psychometric properties of the German version of the OMS-HC scale. The internal consistency of the OMS-HC was good for the total score and satisfactory for two subscales. The fact that the four-item Disclosure subscale showed poor internal consistency might be related to its small item number, which generally is linked to lower alpha values [26]. The internal consistency in our study was similar to the original study [15] regarding the total score, but slightly poorer with respect to the subscale scores.

The factor loadings of items in our exploratory factor analysis suggested that two items (8 and 13) loaded more on the Social Distance factor 3 than on factor 1 or 2, respectively, to which they belonged in the original factor solution. A possible explanation is that item 8 is reverse-scored which may have led to some incorrect answers [27]. Another explanation are contextual, cultural and healthcare system differences between Canada and European countries like Germany and Switzerland regarding the concept of friendship (item 8) [28]. Regarding item 13 (advocacy for people with mental health problems), the concept of advocacy may be more intuitive in Canada than it is in German speaking countries and advocacy for people with mental illness could be shaped by conflictual experiences of MHP [29,31]. The working environment of participants in acute-care psychiatric settings can be characterized by coercion and involuntary admission, which causes emotional reactions in the patients, such as shame and self-stigma [32] and can impair the therapeutic working relationship [33]. Based on these assumptions and given that the two items may cover a unique aspect of the construct, we would recommend to leave them in the scale. However, it remains to be examined in further research whether a more heterogenous composition of the population (HCPs outside of acute-care psychiatric settings) affects the psychometric properties of these two items.

The confirmatory factor analysis showed good model fit with respect to the original version. This is in line with a recent OMS-HC validation in Chile and partly consistent with a Hungarian study that ran a series of factor analyses, resulting in a final two-factor solution [34, 35].

## Strengths and limitations

This study provides the first psychometric evaluation of the German version of the OMS-HC. Participants were recruited in two different countries, representing data from two different health-care systems. A key limitation of this study is the non-representative sample of participants, mainly recruited from the acute-care inpatient psychiatric sector as a convenience sample that is not representative for all healthcare professionals. Various studies have shown differences in attitudes between professionals in outpatient versus inpatient settings [36,38] and psychiatric versus non-psychiatric healthcare systems [2]. Finally, our cross-sectional data did not allow us to assess retest reliability or sensitivity to change during an intervention.

## Conclusion

On the basis of our findings, the OMS-HC can be recommended to assess attitudes of mental health professionals towards people with mental illness and can be usefully applied to effectively develop and evaluate anti-stigma workshops and campaigns in healthcare settings. However, it is advisable to conduct further psychometric tests on a more diverse sample with an emphasis on testing sensitivity to change.

## Supplementary Information

**Additional file 1**. The original English version of the Opening Minds Stigma Scale for Health Care Providers (OMS-HC-15)

**Additional file 2**. The German translation of the Opening Minds Stigma Scale for Health Care Providers (OMS-HC)

## Data Availability

The datasets used and/or analyzed during the current study are available from the corresponding author on reasonable request.
