# Supplementary material for: Psychometric evaluation of the German version of the Opening Minds Stigma Scale for Health Care Providers (OMS-HC)
Source: BMC Psychol. 2021 May 21;9:86. doi: 10.1186/s40359-021-00592-9 (PMC8139058; doi:10.1186/s40359-021-00592-9)
Supplement: Supplementary file 1 — Additional file 1. The original English version of the Opening Minds Stigma Scale for Health Care Providers (OMS-HC-15) [file 40359_2021_592_MOESM1_ESM.pdf]

Psychometric evaluation of the German version of the Opening Minds Stigma Scale for Health Care Providers (OMS-HC)

Gianfranco Zuaboni*[1], Timon Elmer[2], Franziska Rabenschlag[3], Kolja Heumann[4], Susanne Jaeger[5], Bernd Kozel[6], Candelaria I. Mahlke[7], Dominik Schori[8], Anastasia Theodoridou[9], Matthias Jaeger[9,10], and Nicolas Rüsch[11]

Address: [1]Sanatorium Kilchberg, Psychiatric and Psychotherapy Hospital, Switzerland, [2]University of Groningen Netherlands, [3]Psychiatric University Clinics Basel, Switzerland, [4]Department of Psychiatry and Psychotherapy, Medical University Brandenburg, Neuruppin, Germany, [5]Department of Psychiatry and Psychotherapy I, Ulm University, ZfP Südwürttemberg, Germany, [6]University Psychiatric Services Bern, Switzerland, [7]Department of Psychiatry and Psychotherapy, University Medical Centre Hamburg-Eppendorf (UKE), Germany, [8]Directorate of Nursing, Therapies and Social Work, University Hospital of Psychiatry Zurich, Switzerland, [9]Department of Psychiatry, Psychotherapy & Psychosomatics, University Hospital of Psychiatry Zurich, Switzerland, [10]Psychiatrie Baselland, Liestal, Switzerland, [11]Department of Psychiatry and Psychotherapy II, Ulm University and BKH Günzburg, Germany

Email: Gianfranco Zuaboni* - g.zuaboni@sanatorium-kilchberg.ch; Timon Elmer – t.elmer@rug.nl; Franziska Rabenschlag - franziska.rabenschlag@upk.ch; Kolja Heumann - kolja.heumann@mhb-fontane.de; Susanne Jaeger - susanne.jaeger@zfp-zentrum.de; Bernd Kozel - bernd.kozel@upd.ch; Candelaria I. Mahlke - c.mahlke@uke.de; Dominik Schori - mail@dschori.net; Anastasia Theodoridou - anastasia.theodoridou@pukzh.ch; Matthias Jaeger – matthias.jaeger@pbl.ch; Nicolas Rüsch - nicolas.ruesch@uni-ulm.de

*Correspondence:

g.zuaboni@sanatorium-kilchberg.ch

Sanatorium Kilchberg AG

Alte Landstrasse 70, 8802 Kilchberg, Switzerland

Opening Minds Scale for Health Care Providers (OMS-HC-15)*

Mental Health Commission of Canada | Commission de la santé mentale du Canada

These questions ask you to agree or disagree with a series of statements about mental illness. There is no correct answer. Please mark the box that best fits your opinion.

	Strongly Disagree	Disagree	Neither Agree nor Disagree	Agree	Strongly Agree
1. I am more comfortable helping a person who has a physical illness than I am helping a person who has a mental illness.	☐	☐	☐	☐	☐
2. If a colleague with whom I work told me they had a mental illness, I would be just as willing to work with him/her.	☐	☐	☐	☐	☐
3. If I were under treatment for a mental illness I would not disclose this to any of my colleagues.	☐	☐	☐	☐	☐
4. I would see myself as weak if I had a mental illness and could not fix it myself.	☐	☐	☐	☐	☐
5. I would be reluctant to seek help if I had a mental illness.	☐	☐	☐	☐	☐
6. Employers should hire a person with a managed mental illness if he/she is the best person for the job.	☐	☐	☐	☐	☐
7. I would still go to a physician if I knew that the physician had been treated for a mental illness.	☐	☐	☐	☐	☐
8. If I had a mental illness, I would tell my friends.	☐	☐	☐	☐	☐
9. Despite my professional beliefs, I have negative reactions towards people who have mental illness.	☐	☐	☐	☐	☐
10. There is little I can do to help people with mental illness.	☐	☐	☐	☐	☐
11. More than half of people with mental illness don’t try hard enough to get better.	☐	☐	☐	☐	☐
12. I would not want a person with a mental illness, even if it were appropriately managed, to work with children.	☐	☐	☐	☐	☐
13. Healthcare providers do not need to be advocates for people with mental illness.	☐	☐	☐	☐	☐
14. I would not mind if a person with a mental illness lived next door to me.	☐	☐	☐	☐	☐
15. I struggle to feel compassion for a person with mental illness.	☐	☐	☐	☐	☐

*Modgill G, Patten SB, Knaak S, Kassam A, Szeto AC. Opening minds stigma scale for healthcare providers (OMS-HC): Examination of psychometric properties and responsiveness. *BMC Psychiatry* 2014; 14(1):120. http://www.biomedcentral.com/1471-244X/14/120.

*Kassam A, Papish A, Modgill G, Patten S. The development and psychometric properties of a new scale to measure mental illness related stigma by health care providers: The opening minds scale for Health Care Providers (OMS-HC). BMC Psychiatry 2012; 12:62. DOI: 10.1186/1471-244X-12-62.
