# Supplementary material for: Psychometric evaluation of the German version of the Opening Minds Stigma Scale for Health Care Providers (OMS-HC)
Source: BMC Psychol. 2021 May 21;9:86. doi: 10.1186/s40359-021-00592-9 (PMC8139058; doi:10.1186/s40359-021-00592-9)
Supplement: Supplementary file 2 — Additional file 2. The German translation of the Opening Minds Stigma Scale for Health Care Providers (OMS-HC) [file 40359_2021_592_MOESM2_ESM.pdf]

Psychometric evaluation of the German version of the Opening Minds Stigma Scale for Health Care Providers (OMS-HC)

Gianfranco Zuaboni*[1], Timon Elmer[2], Franziska Rabenschlag[3], Kolja Heumann[4], Susanne Jaeger[5], Bernd Kozel[6], Candelaria I. Mahlke[7], Dominik Schori[8], Anastasia Theodoridou[9], Matthias Jaeger[9,10], and Nicolas Rüsch[11]

Address: [1]Sanatorium Kilchberg, Psychiatric and Psychotherapy Hospital, Switzerland, [2]University of Groningen Netherlands, [3]Psychiatric University Clinics Basel, Switzerland, [4]Department of Psychiatry and Psychotherapy, Medical University Brandenburg, Neuruppin, Germany, [5]Department of Psychiatry and Psychotherapy I, Ulm University, ZfP Südwürttemberg, Germany, [6]University Psychiatric Services Bern, Switzerland, [7]Department of Psychiatry and Psychotherapy, University Medical Centre Hamburg-Eppendorf (UKE), Germany, [8]Directorate of Nursing, Therapies and Social Work, University Hospital of Psychiatry Zurich, Switzerland, [9]Department of Psychiatry, Psychotherapy & Psychosomatics, University Hospital of Psychiatry Zurich, Switzerland, [10]Psychiatrie Baselland, Liestal, Switzerland, [11]Department of Psychiatry and Psychotherapy II, Ulm University and BKH Günzburg, Germany

Email: Gianfranco Zuaboni* - g.zuaboni@sanatorium-kilchberg.ch; Timon Elmer – t.elmer@rug.nl; Franziska Rabenschlag - franziska.rabenschlag@upk.ch; Kolja Heumann - kolja.heumann@mhb-fontane.de; Susanne Jaeger - susanne.jaeger@zfp-zentrum.de; Bernd Kozel - bernd.kozel@upd.ch; Candelaria I. Mahlke - c.mahlke@uke.de; Dominik Schori - mail@dschori.net; Anastasia Theodoridou - anastasia.theodoridou@pukzh.ch; Matthias Jaeger – matthias.jaeger@pbl.ch; Nicolas Rüsch - nicolas.ruesch@uni-ulm.de

*Correspondence:

g.zuaboni@sanatorium-kilchberg.ch

Sanatorium Kilchberg AG

Alte Landstrasse 70, 8802 Kilchberg, Switzerland

Unvoreingenommenheit-Skala für psychiatrische Fachpersonen (OMS-HC)[1]

Bitte beantworten Sie alle Fragen, indem Sie jeweils nur ein Kästchen ankreuzen. Vielen Dank!

		Stimme gar nicht zu	Stimme nicht zu	Stimme weder zu noch nicht zu	Stimme zu	Stimme voll zu
1.	Ich fühle mich wohler einer Person zu helfen, die eine körperliche Krankheit hat, als einer Person zu helfen, die eine psychische Krankheit hat.	☐	☐	☐	☐	☐
2.	Wenn ein Kollege*, mit welchem ich arbeite, mir erzählte, er habe eine bewältigte psychische Krankheit, würde ich genauso bereit sein, mit ihm zu arbeiten.	☐	☐	☐	☐	☐
3.	Wenn ich wegen einer psychischen Krankheit in Behandlung wäre, würde ich dies keinem meiner Arbeitskollegen offenbaren.	☐	☐	☐	☐	☐
4.	Ich würde mich selbst für schwach halten, wenn ich eine psychische Krankheit hätte und sie nicht selbst beheben könnte.	☐	☐	☐	☐	☐
5.	Ich würde zögern, Hilfe zu suchen, wenn ich eine psychische Krankheit hätte.	☐	☐	☐	☐	☐
6.	Arbeitgeber sollten eine Person mit einer bewältigten psychischen Krankheit anstellen, wenn sie die beste Person für die Stelle ist.	☐	☐	☐	☐	☐
7.	Ich würde weiter zu einem Arzt gehen, auch wenn ich wüsste, dass der Arzt wegen einer psychischen Krankheit behandelt wurde.	☐	☐	☐	☐	☐
8.	Wenn ich eine psychische Krankheit hätte, würde ich es meinen Freunden erzählen.	☐	☐	☐	☐	☐
9.	Trotz meinen professionellen Überzeugungen reagiere ich negativ auf Menschen, die eine psychische Krankheit haben.	☐	☐	☐	☐	☐
10.	Ich kann wenig tun, um Menschen mit psychischer Krankheit zu helfen.	☐	☐	☐	☐	☐
11.	Die meisten Menschen mit psychischer Erkrankung strengen sich nicht genug an, gesund zu werden.	☐	☐	☐	☐	☐
12.	Ich möchte nicht, dass eine Person mit einer psychischen Krankheit, auch wenn diese angemessen behandelt wäre, mit Kindern arbeitet.	☐	☐	☐	☐	☐
13.	Fachpersonen im Gesundheitsbereich müssen nicht Fürsprecher sein für Menschen mit einer psychischen Erkrankung.	☐	☐	☐	☐	☐
14.	Ich hätte nichts dagegen, wenn eine Person mit einer psychischen Erkrankung neben mir wohnen würde.	☐	☐	☐	☐	☐
15.	Es fällt mir schwer, für eine Person mit einer psychischen Krankheit Mitgefühl zu empfinden.	☐	☐	☐	☐	☐

* Die männliche Schreibweise gilt im gesamten Dokument auch für die weibliche Form

Vielen Dank für Ihre Mitarbeit!

[1] Modgill, G., Patten, S. B., Knaak, S., Kassam, A., & Szeto, A. C. (2014). Opening Minds Stigma Scale for Health Care Providers (OMS-HC): examination of psychometric properties and responsiveness. *BMC Psychiatry, 14*, 120.

[1] Kassam, A., Papish, A., Modgill, G., & Patten, S. (2012). The development and psychometric properties of a new scale to measure mental illness related stigma by health care providers: the Opening Minds Scale for Health Care Providers (OMS-HC). *BMC Psychiatry, 12*, 62.

Deutsche Fassung: Zuaboni, G., Ventling, S., & Rüsch, N. 2015.
